# Supplementary material for: A mobile endocytic network connects clathrin-independent receptor endocytosis to recycling and promotes T cell activation
Source: Nat Commun. 2018 Apr 23;9:1597. doi: 10.1038/s41467-018-04088-w (PMC5913236; doi:10.1038/s41467-018-04088-w)
Supplement: Supplementary file 1 — Supplementary Information [file 41467_2018_4088_MOESM1_ESM.pdf]

A mobile endocytic network connects clathrin-independent receptor endocytosis to recycling and promotes T cell activation.

E.B. Compeer, *et al.*

SUPPLEMENTARY INFORMATION

Supplemental Figure 1

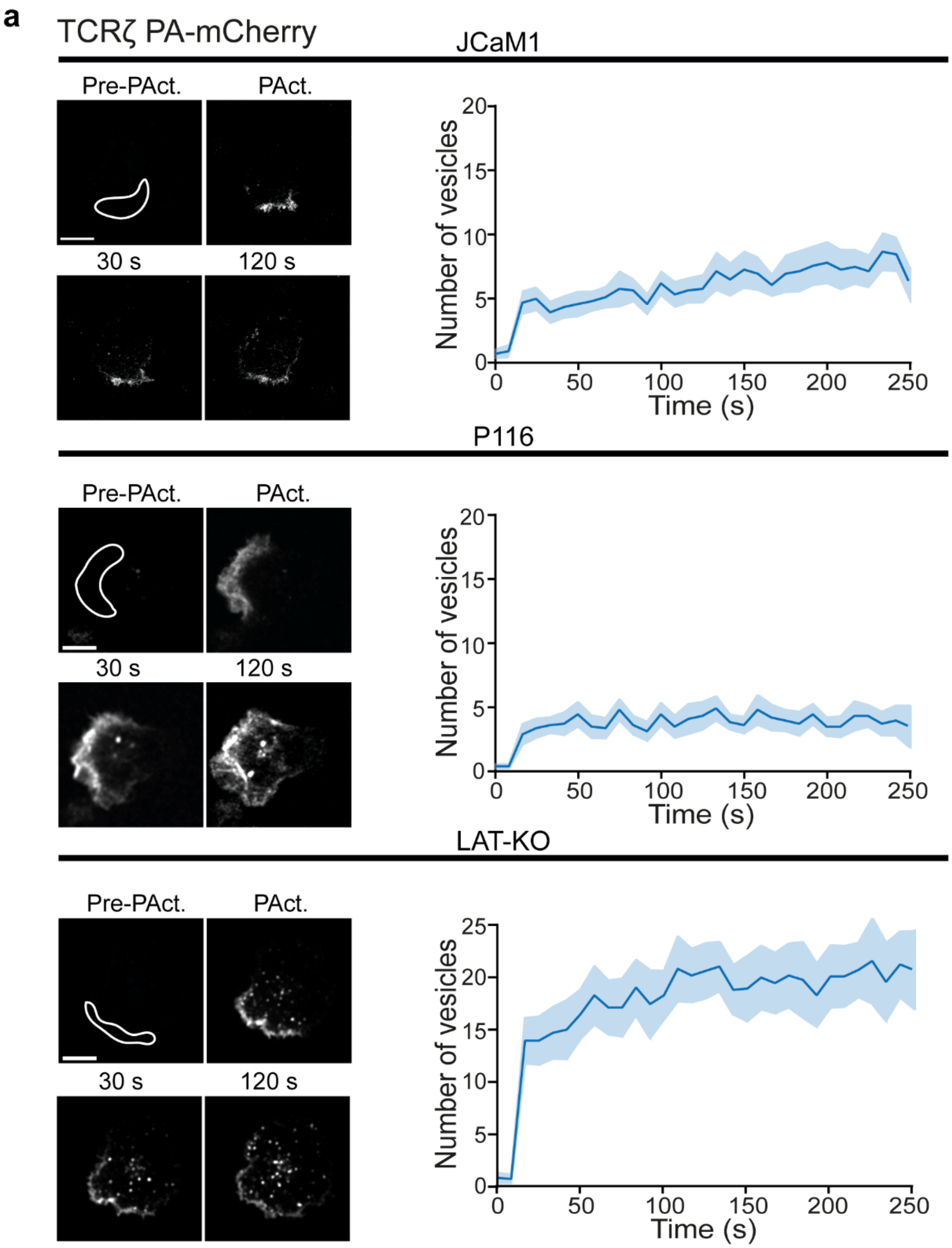

Supplementary Figure 1. Internalization of TCRζ in T cells lacking components of early TCR-signaling pathway. **a** Left: example of TCRζ-PAmCherry expressing T-cells lacking Lck; JCaM1, Zap70; P116, Lat; LAT KO, adhering on activating (anti-CD3ε and anti-CD28) glass surface, photoactivated on outer membrane region of interest, and subsequently imaged for 250 sec. Right: number of PA-mCherry vesicles detected in each frame during the time of acquisition. Scale bars, 5 μm. All analyzed data is obtained from 3 or more independent experiments, with at least 5 cells per experiment. Solid lines represent the mean, surrounding shading areas show ±SEM.

Supplemental Figure 2

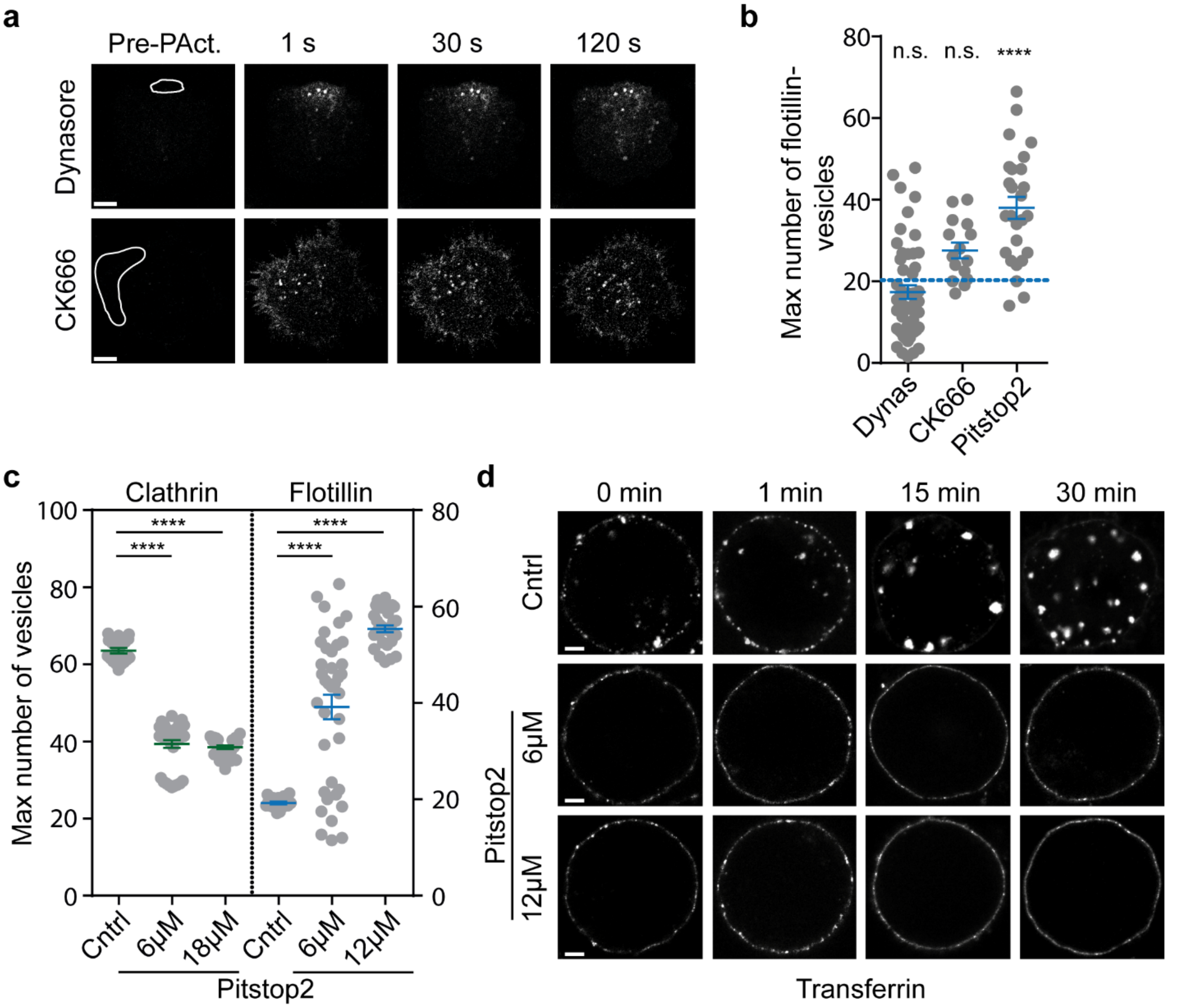

Supplementary Figure 2. **Effect of inhibitors on TCRζ, clathrin, flotillin and transferrin internalization.**. **a** Example of TCRζ-PAmCherry expressing T cells treated with Dynasore (top) and CK666 (bottom), adhering on activating glass surface, photoactivated on outer membrane region of interest, and subsequently imaged for 250 sec. and **b** the number of PA-mCherry vesicles detected in each frame during the time of acquisition of T-cells treated with Dynasore, CK666, and Pitstop2. Each dot represents a cell. Data obtained from 3 independent experiments. **c** Maximal number of (left) Clathrin- and (right) Flotillin- PAmCherry vesicles detected during the time of acquisition of T cells treated with indicated concentration of Pitstop2. Each dot represents a cell. Data obtained from 3 independent experiments. **d** Representative images of Jurkat T cells treated with 6 or 12 μM of Pistop2, incubated with transferrin-alexa488 and fixed at the indicated times. Images are representative of 2 independent experiments. (±SEM). ns, not significant; **** $p < 0.00001$; Mann-Whitney t-test. Scale bars, 2 μm.

Supplemental Figure 3

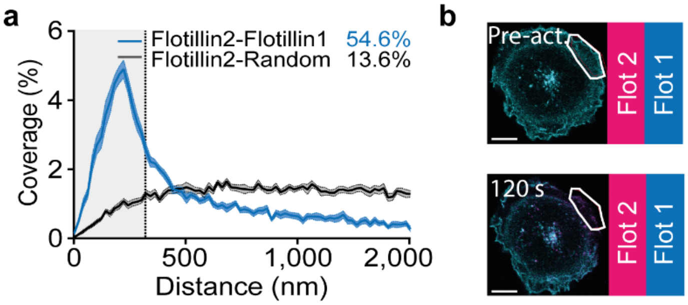

Supplementary Figure 3. **Cross-channel nearest neighbour analysis of flotillin 1 and 2**. **a** Cross-channel nearest neighbor analysis between vesicles defined by Flotillin1-eGFP and Flotillin2-PAmCherry in Jurkat T cells activated on anti-CD3ε and anti-CD28 coated surfaces. **b** Examples of Flotillin1eGFP and Flotillin2-PAmCherry expressing T-cells adhering on activating glass surface, photoactivated on outer membrane region of interest as indicated. Data obtained from 4 independent experiments. Solid lines represent the means, surrounding doted lines show ±SEM Scale bars, 2 µm.

Supplemental Figure 4

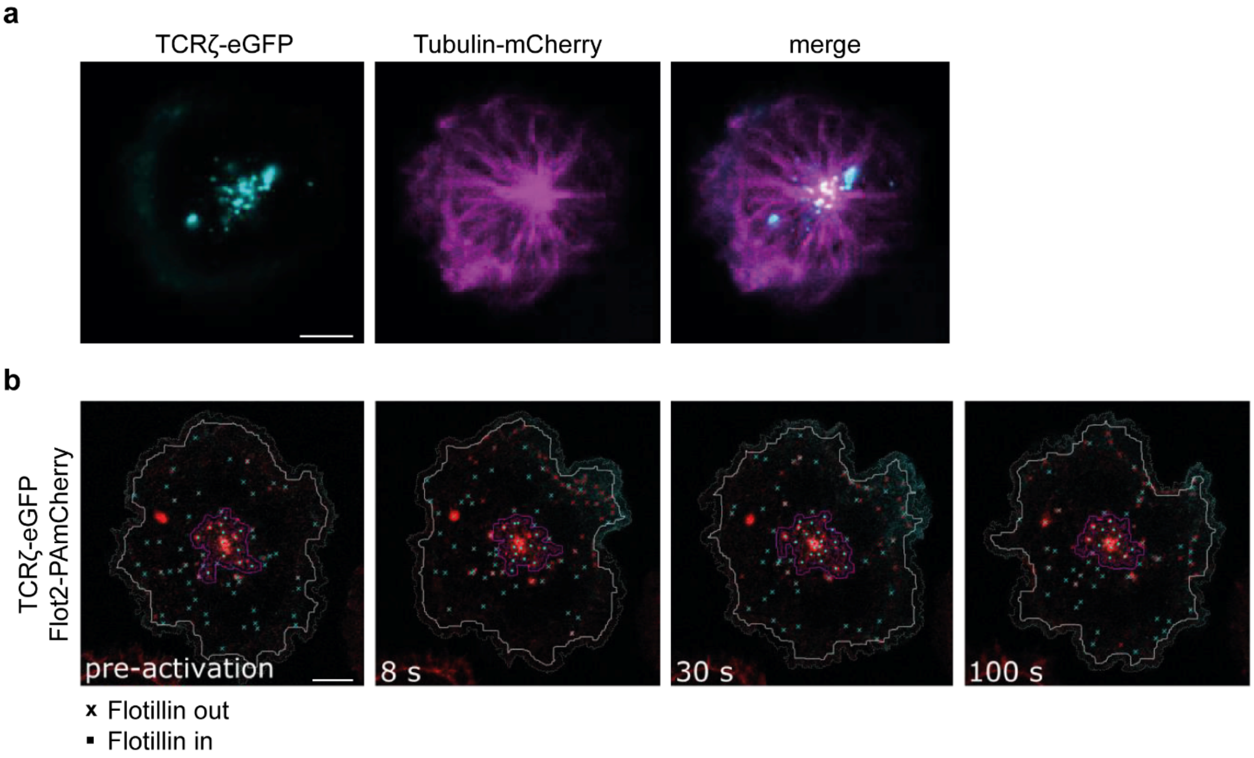

Supplementary Figure 4 Central TCRζ endosomes co-localize with microtubule-organizing centre in activated T cells. a TCRζ-EGFP and ßTubulin-mCherry expressing T cells on activating (anti-CD3 and CD28 coated) glass surface. Images representative of 2 independent experiments **b** Example of detection of vesicles observed within the central endosomal region and out of it. Scale bars, 5 μm.

Supplemental Figure 5

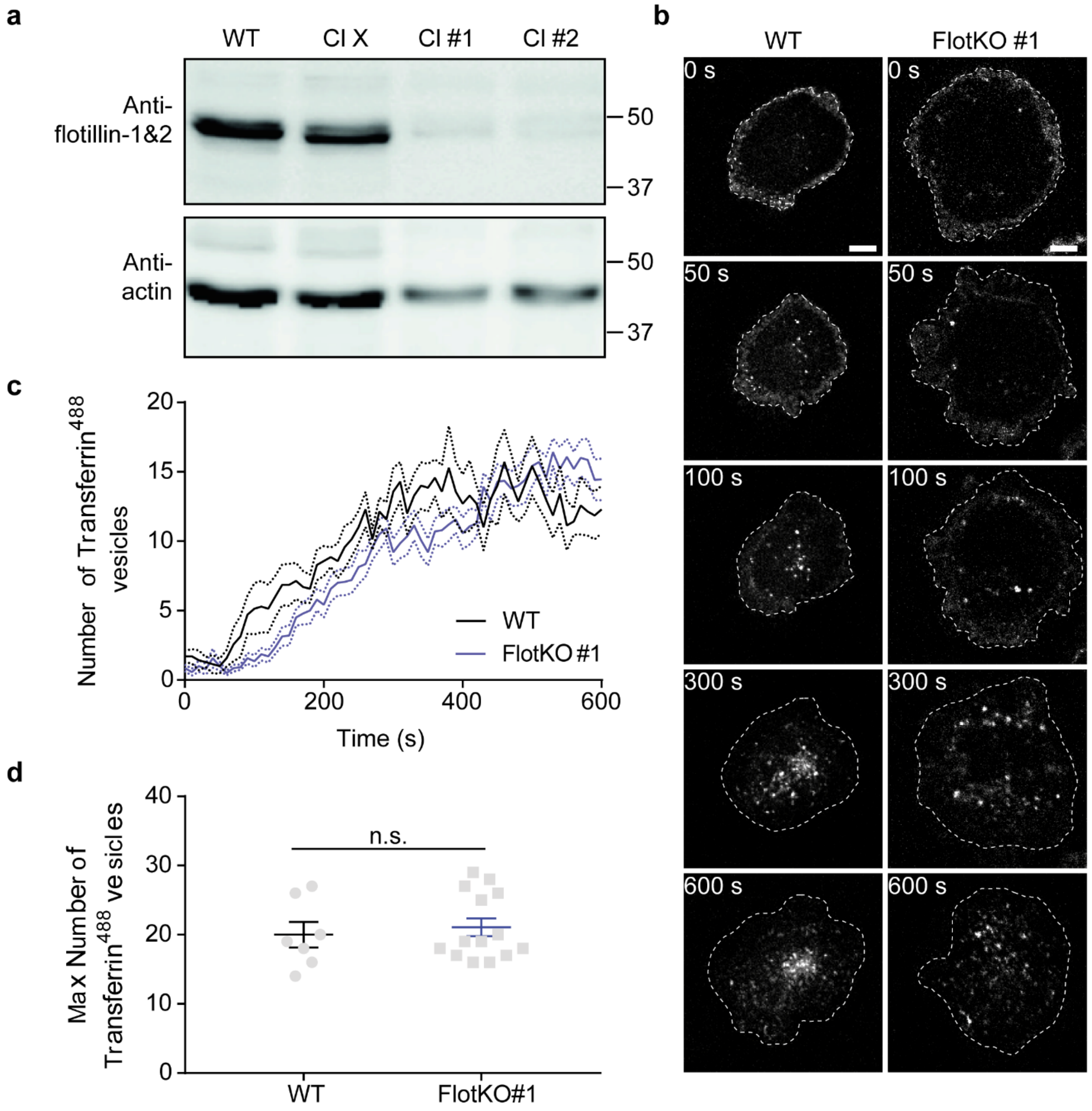

Supplementary Figure 5. Flotillin expression in Flotillin KO jurkat T cells and effects of the KO on transferrin internalization. a Western blot probed with antibodies against flotillin 1 and 2 in wildtype jurkat (first lane) showing unsuccessful CRISPR/Cas9 gene edition (second lane) or successful (right two lanes) knock out flotillin 1 and 2. Actin was used as loading control. **b** Representative live images of WT and flotillin1/2 KO cells incubated with transferrin-alexa-488 and allowed to internalize it for the indicated times. Scale bar, 2 µm. **c, d** Number of Alexa488 positive vesicles detected in each frame during the time of acquisition. Solid lines show the mean, surrounding doted lines show ±SEM d Maximum number of Alexa488 positive vesicles detected in a given frame during the 600 sec acquisition time. Each dot represents a cell. All data is obtained from 3 independent experiments. Small horizontal lines indicate mean (±SEM). ns, not significant; Mann-Whitney t-test.

Supplemental Figure 6

a

b

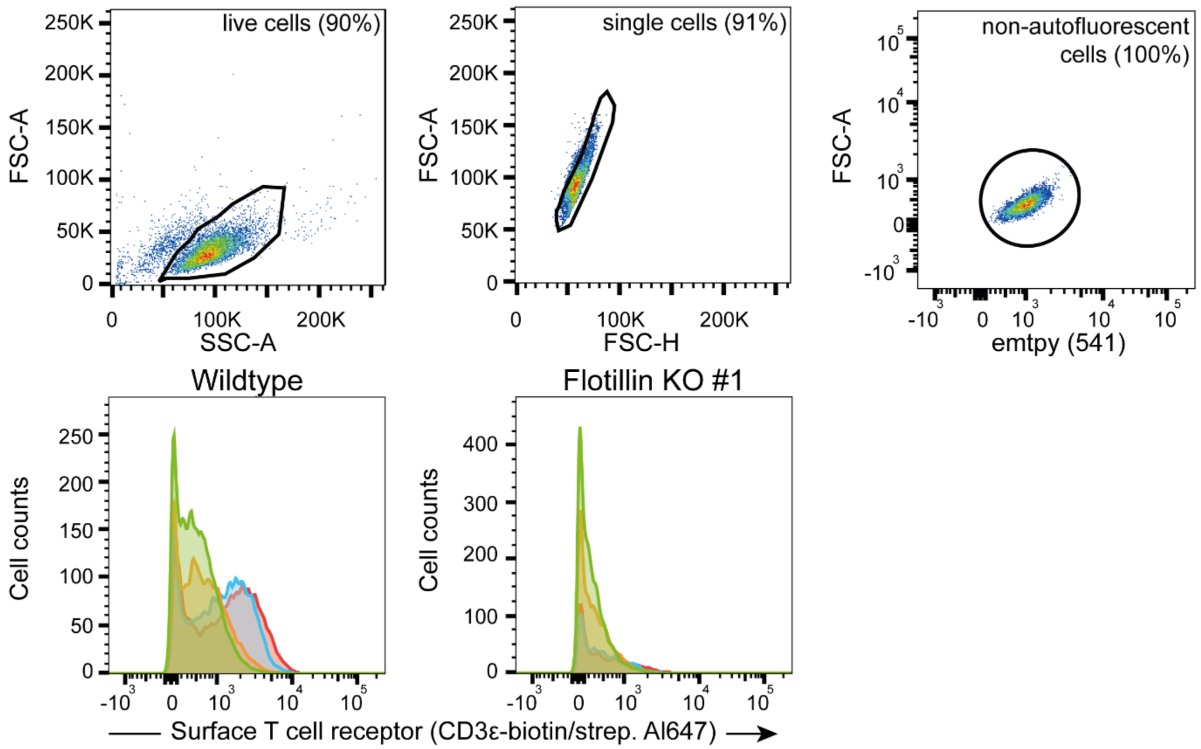

Supplementary Figure 6. **Flow cytometry gating strategy. a** FSC and SSC dot plots were used to identify live cells, doublets were eliminated by using a pulse geometry gate with FSC-H and FSC-A and autofluorescence in a dump gate was used to eliminate any other non-live and single cells. **b** Geometric Mean Fluorescent Intensity of 660nm was determined to quantify surface TCR complexes upon T cell activation by labelling with biotinylated anti-CD3ε and Alexa647-conjugated streptavidin.

Supplemental Figure 7

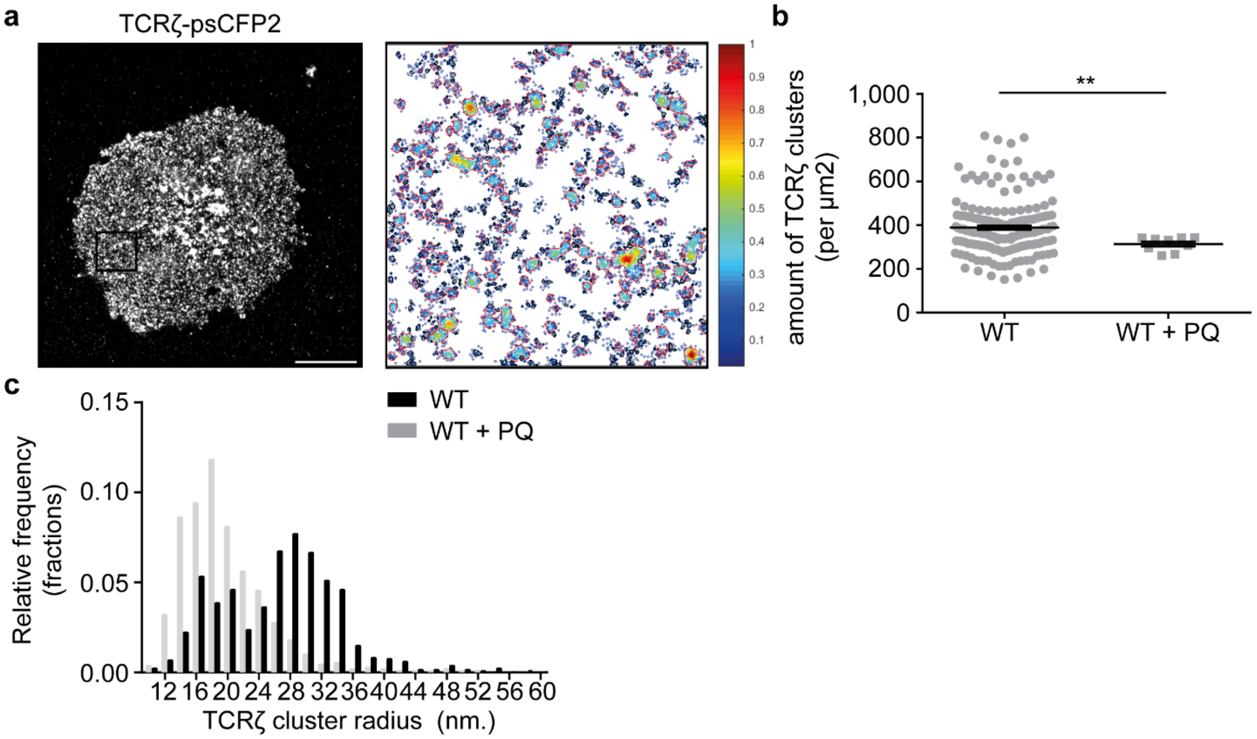

Supplementary Figure 7. Pharmacological inhibition of recycling affects TCRζ nanoscale spatial organization in activated T cells. **a** Single-molecule images of TCRζ -PSCFP2 in Jurkat T cells on activating (anti-CD3ε and anti-CD28 antibodies) coated glass surfaces after pre-treatment with recycling inhibitor primaquine. Left: density map with TCRζ -PSCFP2 clusters identified by DB-SCAN, per 3 µm × 3 µm region in primaquine treated T cells. Each dot represents a cell. Scale bars, 5 µm. **b** Number of TCRζ -PSCFP2 clusters identified by DB-SCAN, per 3 µm × 3 µm region in activated untreated (left) and primaquine treated (right) T-cells. **c** Size of radius of TCRζ -PSCFP2 clusters in activated untreated (grey bars) and primaquine treated (black bars) T-cells. c Right: All analyzed data is obtained from 2 independent experiments. Error bars indicate mean (±SEM). ns, not significant; ** $p < 0.001$; Mann-Whitney t-test.

Supplemental Figure 8

a

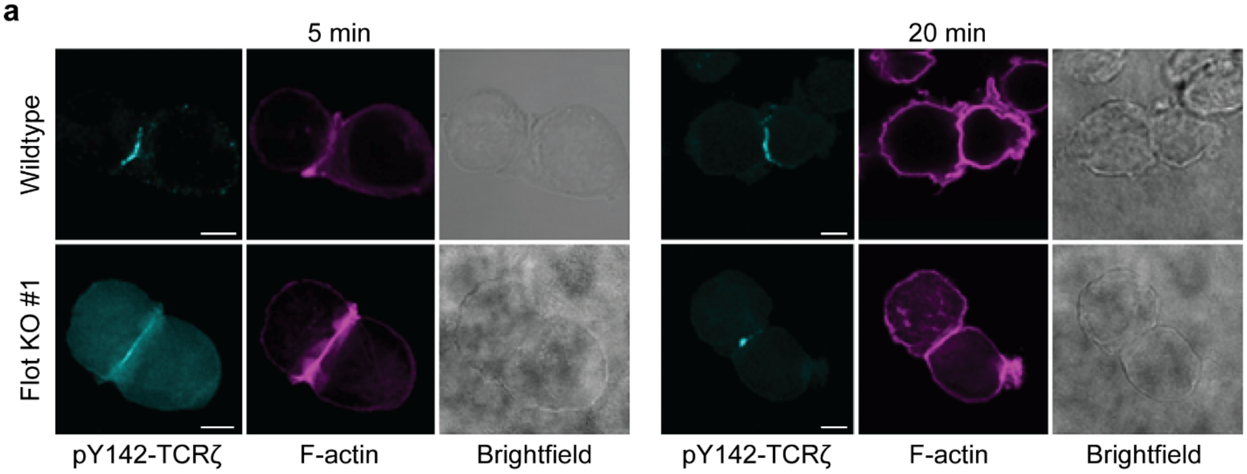

b

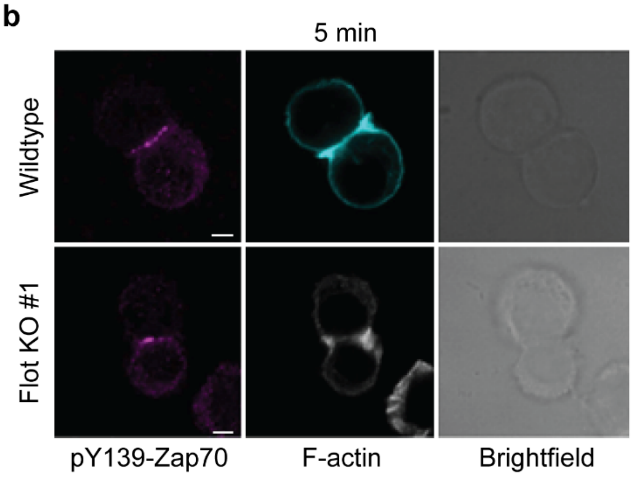

Supplementary Figure 8. **Flotillin1/2 knock-out partially impairs TCRζ and Zap70 phosphorylation in Jurkat T cells activated by SEE-pulsed Raji B cells**. WT or flotillin1/2 KO Jurkat T cells were allowed to form conjugates for 5 or 10 min with SEE-pulsed Raji B cells, fixed and stained with phalloidin and **a** an antibody against phosphorylated TCRζ or **b** against phosphorylated Zap70. There was no signal for pZap70 at 20 min after activation. Images taken with the same settings for a given condition and representative of 3 independent experiments. Scale bar, 5 μm.

Supplemental Figure 9

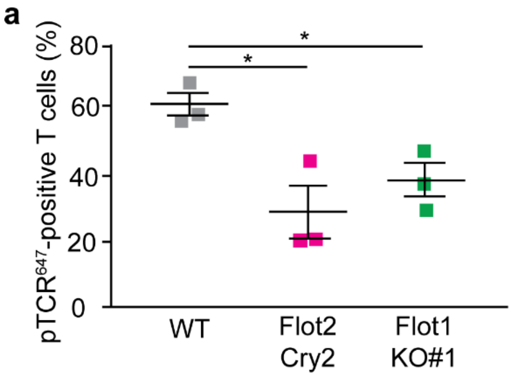

Supplementary Figure 9. **Artificially aggregating flotillin positive endosomes impairs T cell activation**. **a** Untransfected WT, WT transfected with Flotillin2-ECFP-CIB1 and flotillin2-mCerulean-Cry2Clust or flotillin1/2 KO Jurkat T cells were activated with anti CD3 and CD28 for 5 min, fixed and the percentage of cells positive for phosphorylated TCR was determined by flow cytometry in 3 independent experiments. Error bars indicate mean (±SEM). * $p < 0.05$; Mann-Whitney t-test.

Supplemental Figure 10

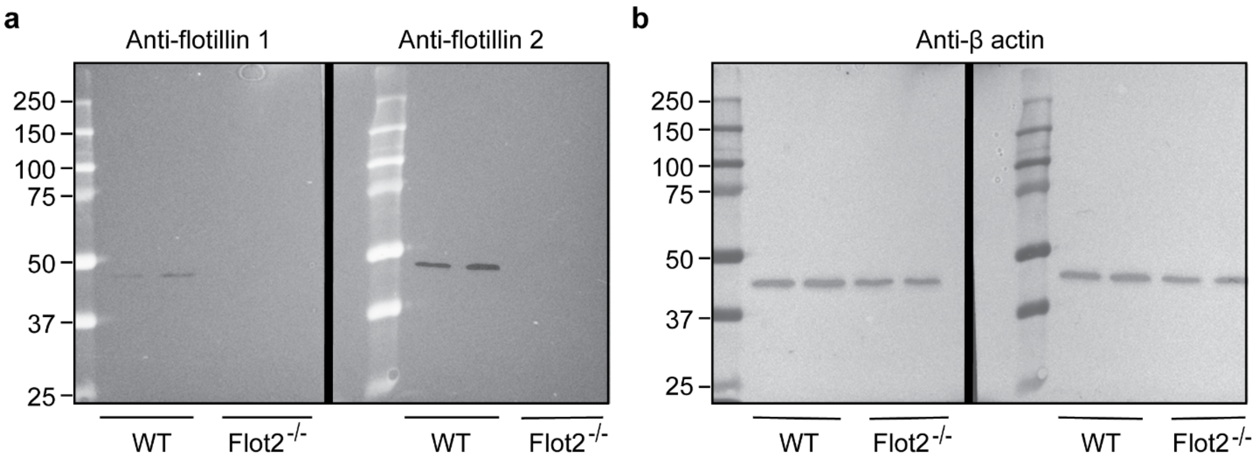

Supplementary Figure 10. **Whole scans of blots from experiments of Figure 8a**. **a** Blots stained with antibodies against Flotillin 1 and 2, and **b** against β-actin as loading control. Molecular weights as indicated.
