# Supplementary material for: A mobile endocytic network connects clathrin-independent receptor endocytosis to recycling and promotes T cell activation
Source: Nat Commun. 2018 Apr 23;9:1597. doi: 10.1038/s41467-018-04088-w (PMC5913236; doi:10.1038/s41467-018-04088-w)
Supplement: Supplementary file 3 — Description of Additional Supplementary Files [file 41467_2018_4088_MOESM3_ESM.pdf]

Description of Additional Supplementary Files

File Name: Supplementary Movie 1
Description: Jurkat T cells expressing TCRζ-PAmCherry, allowed to adhere on Poly-LLysine coated surfaces, photoactivated on a region of interest and subsequently imaged for 250 sec.

File Name: Supplementary Movie 2
Description: Jurkat T cells expressing TCRζ-PAmCherry, activated on anti-CD3 and antiCD28 coated surfaces, photoactivated on a region of interest and subsequently imaged for 250 sec.

File Name: Supplementary Movie 3
Description: Jurkat T cells expressing Lck-PAmCherry, activated on anti-CD3 and antiCD28 coated surfaces, photoactivated on a region of interest and subsequently imaged for 250 sec.

File Name: Supplementary Movie 4
Description: Flotillin1/2 KO Jurkat T cells expressing TCRζ-PAmCherry, activated on antiCD3 and anti-CD28 coated surfaces, photoactivated on a region of interest and subsequently imaged for 250 sec.
